# Boosting Mouse Defense against Lethal *Toxoplasma gondii* Infection with Full-Length and Soluble SAG1 Recombinant Protein

**DOI:** 10.3390/vaccines11111678

**Published:** 2023-11-02

**Authors:** Xiang Li, Wei Yuan, Ting He, Ruiying Guo, Xiuxian Du, Yanhong He, Xuan Li, Saeed El-Ashram, Ebtesam M. Al-Olayan, Na Yang, Xiaoyu Sang

**Affiliations:** 1 Key Laboratory of Livestock Infectious Diseases, Shenyang Agricultural University, Ministry of Education, Shenyang 110866, China; lx19980405@163.com (X.L.); yuanwei110923@163.com (W.Y.); 2021240709@stu.syau.edu.cn (T.H.); fruiming@stu.syau.edu.cn (R.G.); 2021220598@stu.syau.edu.cn (X.D.); 2022220595@stu.syau.edu.cn (Y.H.); 13081305895@163.com (X.L.); 2 College of Animal Science and Veterinary Medicine, Shenyang Agricultural University, Shenyang 110866, China; 3 Zoology Department, Faculty of Science, Kafrelsheikh University, Kafr El-Sheikh 33516, Egypt; saeed_elashram@yahoo.com; 4 Department of Zoology, College of Science, King Saud University, Riyadh 11451, Saudi Arabia; eolayan@ksu.edu.sa

**Keywords:** *Toxoplasma gondii*, SAG1, full-length, defense, mouse

## Abstract

Toxoplasmosis is a major worldwide protozoan zoonosis. The surface antigen 1 (SAG1) of *Toxoplasma gondii* (*T. gondii*) has always been recognized as an ideal vaccine candidate antigen. However, the intact and soluble SAG1 protein is usually difficult to acquire in vitro, which is unfavorable for employing the recombinant protein as a vaccine candidate antigen. In the present study, we obtained the full-length SAG1 recombinant protein in soluble form by *Escherichia coli* Transetta (DE3) cells under optimized expression conditions. The immunogenicity and protective ability of this recombinant protein against *T. gondii* acute infection were evaluated in a mouse model. Monitoring changes in serum antibody levels and types, the presence of cytokines, and the rate of lymphocyte proliferation in vaccinated mice were used to assess humoral and cellular immune responses. Additional assessments were performed to determine the protective potency of the recombinant protein in combating *T. gondii* RH tachyzoites. It was found that the titers of both IgG2a and IgG2b were considerably greater in the immunized mice compared to the titers of IgG1 and IgG3. The levels of Th1-type cytokines (IFN-γ, IL-12p70, IL-2, and TNF-α) and Th2-type cytokines (IL-10) significantly increased when splenocytes from immunological group mice were treated with *T. gondii* lysate antigen. Compared to the control group, a recombinant protein substantially increased the longevity of infected mice, with an average death time prolonged by 14.50 ± 0.34 days (*p* < 0.0001). These findings suggest that the full-length and soluble SAG1 recombinant protein produced potent immune responses in mice and could be a preferred subunit vaccine candidate for *T. gondii*, offering a feasible option for vaccination against acute toxoplasmosis.

## 1. Introduction

Toxoplasmosis is a prevalent parasitic disease attributed to the protozoan *Toxoplasma gondii* (*T. gondii*) [1]. It is one of the most common zoonotic infections globally, affecting 30–50% of the population. *T. gondii* has an extensive range of intermediate hosts, but only felids, like cats, are definitive hosts that release infectious oocysts into the environment [2]. *T. gondii* tachyzoites can almost invade all nucleated cells, including some immune cells, such as macrophages and dendritic cells. Infected macrophages and dendritic cells, employed as “Trojan horses”, contribute to transmitting this parasite in the host body [3]. The infection of *T. gondii* is usually asymptomatic in immunocompetent individuals. However, it can sometimes be serious, even fatal, for immunodeficient individuals, such as AIDS patients and pregnant women [4,5,6]. *T. gondii* infection during the first trimester of pregnancy may result in abortion, early delivery, stillbirth, or fetal malformations in a few cases. *T. gondii* infection throughout pregnancy’s second and third trimesters can result in lesions or malformations of the fetal brain, eyes, liver, heart, lungs, and other organs [7,8]. Furthermore, animal toxoplasmosis also brings unignorable economic losses to the livestock industry, especially by causing fetal death and stillbirths in all types of livestock, particularly pigs, sheep, and goats [9]. Sulfonamides are effective in treating acute toxoplasmosis but have limited efficacy in chronic toxoplasmosis. Additionally, the side effects of sulfonamide drugs should be considered when prescribing them [10]. The major routes of human infection by *T. gondii* include ingesting uncooked food or water contaminated with the parasite’s cyst or oocyst. These sources of infection are closely associated with animal toxoplasmosis [11,12].

Developing a vaccine against animal toxoplasmosis is important for preventing and controlling human toxoplasmosis. Until now, no commercial vaccine is available to prevent human toxoplasmosis. However, in the European market, Toxovax, an animal *Toxoplasma* vaccine, has been approved to avoid sheep abortion. This vaccine was prepared from strain S48 of *T. gondii*, which was initially isolated from a sheep fetus that had miscarried in New Zealand. The strain underwent 3000 generations of laboratory passages to obtain a weakly virulent strain. This strain was avirulent in mice, unable to form tissue cysts or oocysts [13]. However, due to the incomplete understanding of the molecular basis of weak strain S48 tachyzoites and the possibility of spontaneous mutations causing the weak strain to regain its virulence at any time, this vaccine is no longer used in the market. With their advantages and deficiencies in recent decades, *T. gondii* vaccines prepared through genetic engineering technology, including subunit, nucleic acid, nanoparticle, and gene knockout strains, have been extensively and deeply explored [14]. Researchers have screened and evaluated many protective antigens among the *T. gondii* tachyzoite invasion and pathogenesis-related proteins. Surface antigen 1 (SAG1), as a major ligand for cell attachment, is specifically expressed on the surface of tachyzoites and has shown superior immunogenicity and protective ability [15]. Further, the sequence of SAG1 was highly conserved among three main clonal lineages, types I (pathogenic and lethal in mice) and type II/III (cystic type). The development of a subunit vaccine based on the SAG1 protein is continuously being explored, including purified natural SAG1, recombinant SAG1 produced by different expression systems, or SAG1-derived peptides [16,17,18]. However, SAG1 is a highly conformational protein constructed by six disulfide bonds, and its immunogenicity and antigenicity largely depend on the appropriate folding through disulfide bonding [19,20]. Unfortunately, the expression of SAG1 proteins in prokaryotic cells usually results in inactive inclusion bodies [21,22]. The denaturation and renaturation of inclusion bodies can take a long time and, importantly, may not necessarily restore the natural conformation of the protein, which may influence its immunogenicity. To overcome this problem, some researchers obtained antigenic SAG1 protein by fusing it with solubility/folding enhancing proteins such as thioredoxin or glutathione-S-transferases (GST) or by truncating its expression in *Escherichia coli* (*E. coli*), which are averse to preparing the antigen for a subunit vaccine [23,24,25]. 

In the present study, under optimized expression conditions, full-length and soluble SAG1 recombinant protein was obtained by the *E. coli* Transetta (DE3) cells, which could supplement the rare codon of the *SAG1* gene. As an immunogen, the purified recombinant protein combined with Freund’s complete adjuvant/incomplete adjuvant stimulated mice to produce a robust humoral and cellular immune response and significantly prolonged the survival time of immunized mice in the lethal infectious model. These results show that full-length and soluble SAG1 recombinant protein could be an optical immunogen for constructing a subunit vaccine against *T. gondii*.

## 2. Materials and Methods

### 2.1. Mice and Parasites

Female Balb/c mice aged six to seven weeks were obtained from Liaoning Changsheng Biotechnology Company (Shenyang, China). Strict adherence to the animal husbandry rules of Shenyang Agricultural University was followed throughout mouse breeding and assessment. The institutional ethics committee approved the experimental animal research at Shenyang Agricultural University (Permit No. 2022111001). *T. gondii* RH tachyzoites were cultured in Vero cells, and total RNA extraction, *Toxoplasma* lysate antigen (TLA) preparation, and a mouse challenge experiment were all performed using these tachyzoites as previously described [26].

### 2.2. Cloning and Expression of Full-Length SAG1 Recombinant Proteins in Prokaryotic Expression System

The coding sequence (CDS) for *SAG1* gene of *T. gondii* RH strain was obtained by PCR using gene-specific primers (Forward: 5′TCCGGAATTCATGTCGGTTTCGCTG3′; Reverse: 5′ACCGCTCGAGTCAGCGACACAAG3′). The amplicons were cloned into the pET28a vector (Invitrogen, Carlsbad, CA, USA) through EcoRI and XholI enzymes (Transgene Biotech, Beijing, China) digestion and T4 DNA ligase (Transgene Biotech, Beijing, China). The chemically competent *E. coli* Transetta (DE3) cells [genotype: F-ompT hsdSB(rB-mB-)gal dcm lacY1 (DE3) pRARE (argU, argW, ileX, glyT, leuW, proL) (Camr)] (Transgene Biotech, Beijing, China), characterized by supplementing the six lacking codons in *E. coli*, were transformed with the recombinant plasmid. The full-length SAG1 recombinant protein was present in the supernatant of the lysed transformed cells, which had been cultivated for 16 h under induced conditions of 0.1 mM Isopropyl β-d-1-thiogalactopyranoside (IPTG) and 16 °C. The SAG1 recombinant protein was purified using Ni-NTA beads (Transgene Biotech, Beijing, China). A BCA assay kit (Beyotime Biotechnology, Shanghai, China) was utilized to assess the concentration of the SAG1 recombinant protein, and the purified protein was stored at −80 °C.

### 2.3. Mouse Immunization and Challenge with T. gondii Tachyzoite

Female Balb/c mice aged six to seven weeks were divided into two groups, each containing 12 mice. The control group was immunized with an equal volume of PBS mixed with the same adjuvant. For the first immunization, 100 μg of SAG1 recombinant protein was mixed with an equal volume of Freund’s complete adjuvant (Sigma-Aldrich, Louris, MI, USA) and injected subcutaneously into the experimental group. Subsequent three immunizations were administered at 2-week intervals using an equal volume of Freund’s incomplete adjuvant (Sigma-Aldrich, Louris, MO, USA). At 2, 4, 6, and 8 weeks after the first immunization, blood was drawn from the tail veins of the six same mice in each group. Sera were collected and centrifuged at 4000× *g* for 5 min before being kept at −20 °C. Two weeks after the fourth vaccination, nine mice per group were intraperitoneally infected with 50 *T. gondii* RH strain tachyzoites and further randomly divided into two groups; one group of 6 mice was used to observe survival time, and the other group of 3 mice was used to detect cytokine levels in the serum on the seventh day after infection.

### 2.4. Measurement of Humoral Response

Enzyme-linked immunosorbent assay (ELISA) detected, as previously described, anti-Toxoplasma IgG in serum isolated at 2, 4, 6, and 8 weeks after the first immunization, and IgG2a, IgG2b, and IgG3 antibodies in serum samples isolated at the 14th day after the last inoculation. Briefly, microtiter plates were coated with 100 μL (10 μg/mL) TLA diluted in 0.05 M potassium phosphate buffer (pH 8) and incubated overnight at 4 °C. The plates were then washed thrice in phosphate-buffered saline (PBS) supplemented with 0.05% Tween 20 (PBST) before being subjected to a blocking step for 1 h at 37 °C in PBST supplemented with 1% bovine serum albumin (BSA). The plates were washed with PBST and then left to incubate at room temperature for 1 h. The serum was diluted to a 1:100 ratio in PBS during this incubation. Subsequently, the plates were examined utilizing HRP-conjugated goat anti-mouse IgG1, IgG2a, IgG2b, IgG3, or total IgG (ImmunoWay Biotechnology Company, Plano, TX, USA) as secondary antibodies for the purpose of isotype analysis. The measurement of peroxidase activity in each well was conducted by adding 100 μL of TMB (3,3′,5,5′-Tetramethylbenzidine) solution (Beyotime Biotechnology, Shanghai, China). The optical density (OD) was determined at a wavelength of 450 nm utilizing an ELISA microplate reader (Tecan, Männedorf, Switzerland) after terminating the reaction by adding 50 μL of 2 M H_2_SO_4_. Each sample was assessed three times.

### 2.5. Spleen Lymphocyte Proliferation Assay

As previously described, splenocyte suspensions were taken from three mice in each group two weeks after the last immunization [26]. This was performed using the following steps: pushing the spleens through a wire mesh, purifying the suspensions by eliminating the red blood cells (RBCs) using an erythrocyte lysis solution, and resuspending them in 1640 medium with 10% FBS. In 96-well plates, 5 × 10^5^ cells were cultured and enhanced for 48 and 72 h at 37 °C with 5% CO_2_ using TLA (20 µg/mL), concanavalin A (ConA) (CAS number: 11028-71-0) (positive control; 5 µg/mL) (Solarbio, Beijing, China), glutathione-S-transferases (GST) (expressed and purified in *E. coli* in our lab) (20 µg/mL) (negative control), or 1640 medium alone (blank control). After that, 10 µL of cell counting kit-8 (CCK8) (Glpbio, Shanghai, China) was put into each well and incubated for 4 h. The stimulation index (SI) was calculated using the following formula: (OD_450_ stimulation-OD_450_ medium blank): (OD_450_ 1640-OD_450_ medium blank). Each sample was assessed three times.

### 2.6. Cytokine Assays

The splenocytes were cultured in 96-well plates to conduct a lymphocyte proliferation experiment as previously described [26]. These cells were then stimulated with TLA at a 20 µg/mL concentration. Cell-free supernatants were collected at 24, 72, and 96 h and analyzed for the levels of interleukin-2 (IL-2), IL-4, IL-10, IL-12p70, interferon-γ (IFN-γ), and tumor necrosis factor-α (TNF-α) using an ELISA kit (Elabscience, Shanghai, China). The six cytokine concentrations were determined in triplicate. In addition, serum was collected from the three infected mice in each challenge group on the seventh day after infection to assess the cytokines levels in the blood as previously described.

### 2.7. Flow Cytometry Analysis

The proportions of CD4^+^ and CD8^+^ T lymphocytes were determined by flow cytometry as previously described [26]. Splenocyte suspensions taken from three mice used for the spleen lymphocyte proliferation assay were labeled with fluorescently labeled monoclonal antibodies (mAbs), including FITC-conjugated anti-mouse CD45, Brilliant Violet 510TM-conjugated anti-mouse CD3, APC-Cy7-conjugated anti-mouse CD4, and PE-conjugated anti-mouse CD8a (BioLegend, San Diego, CA, USA) for 30 min in the absence of light. Subsequently, the samples were washed three times with cold PBS buffer. The FACS Aria III flow cytometer (BD Biosciences, San Jose, CA, USA) was used to identify and analyze the samples.

### 2.8. Statistical Analysis

For statistical analysis, GraphPad Prism 9.0 was used. The data from the normal distribution were given as mean ± SD. The antibody titer data, cytokines, lymphoproliferative assay, and mean death time were compared using two-way ANOVA test. The threshold for statistical significance was *p* < 0.05, with **** *p* < 0.0001, *** *p* < 0.001, ** *p* < 0.01, and * *p* < 0.05.

## 3. Results

### 3.1. Full-Length SAG1 Recombinant Protein Expression and Purification In Vitro

**Figure 1:**
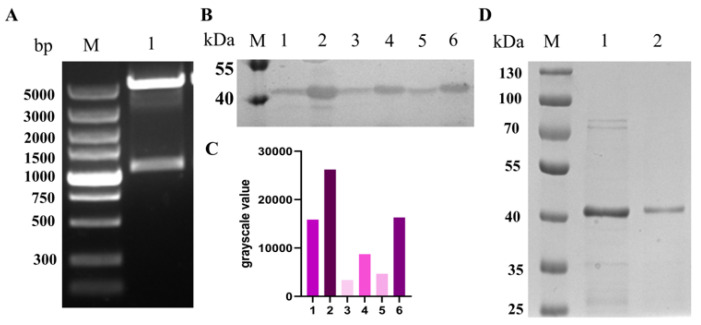
Construction of pET28a-SAG1 recombinant plasmid and expression of full-length SAG1 recombinant protein in soluble form. (**A**) Identification of recombinant pET28a-SAG1 plasmid by EcoRI and XholI enzyme digestion. The lengths of *SAG1* gene and pET28a vector were 1011 bp and 5369 bp, respectively; M: Stander DNA marker. (**B**) Identification of SAG1 recombinant protein expressed in *E. coil* Transetta (DE3) cells by Western blot. 1,3,5: The soluble protein of interest in the cell lysates supernatant from three randomly selected single colonies; 2,4,6: The insoluble protein of interest in cell lysate precipitation from three randomly selected single colonies; M: Stander protein marker. (**C**) The grayscale values of bands in Western blot were analyzed by Image J soft V 1.54d. (**D**) Purified SAG1 recombinant protein identified by SDS-PAGE. 1,2: Target protein was in eluent buffer; M: Stander protein marker.

The intact coding sequence (1011 bp) of the *SAG1* gene was amplified by PCR and inserted into the pET28a vector through EcoRI and XholI enzyme digestion and T4 DNA ligase (Figure 1A). The resulting recombinant plasmid, named pET28a-SAG1, was then transformed into *E. coli* Transetta (DE3) cells, which supplemented rare codons of the SAG1 gene, including AGA, AGG, TGA, and CGA. Three randomly selected monoclonal colonies could all express the SAG1 recombinant protein in a soluble form, as indicated by detecting the desired protein in the cell lysis supernatant (Figure 1B,C). SDS-PAGE analysis was used to characterize the purified SAG1 recombinant protein after it was isolated using affinity chromatography (Figure 1D).

### 3.2. The SAG1 Recombinant Protein Stimulated High Levels of IgG Production in Mice, Including Subtypes IgG1, IgG2a, IgG2b, and IgG3

**Figure 2:**
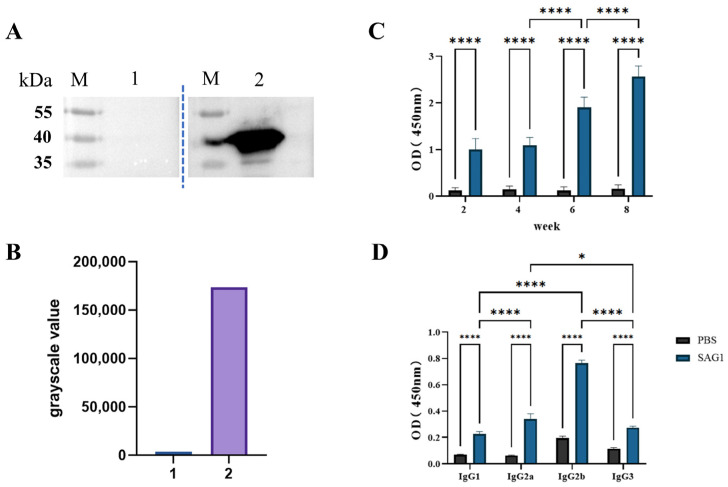
Serum IgG, IgG1, IgG2a, IgG2b, and IgG3 antibody levels in immunized and control mice. (**A**) Western blot analysis for native SAG1 in TLA with the experimental mouse sera. 1: The reaction of TLA with the sera isolated from mice in the control group; 2: The reaction of TLA with the sera isolated from mice in the immunized group; M: Stander protein marker. (**B**) The grayscale values of bands in Western blot were analyzed by Image J soft V 1.54d. (**C**) Detection of IgG antibodies in the serum of Balb/c mice 2, 4, 6, and 8 weeks after the initial vaccination. (**D**) IgG1, IgG2a, IgG2b, and IgG3 antibody detection in immunized mice on the 14th day after the last immunization. Data of mice in each group are shown as mean ± SD (*n* = 6), with statistical significance represented by asterisks (* *p* < 0.05, **** *p* < 0.0001).

Blood was drawn from the tail veins of each set of the six same mice at two, four, six, and eight weeks after the initial vaccination. The serum was isolated for polyclonal antibody detection. As shown in Figure 2A,B, serum isolated from immunized mice specifically recognized the native SAG1 protein in TLA, while the control group did not show any recognition. During the ELISA test, TLA was used as a coated antigen to detect the polyclonal antibody levels in the serum. With multiple immunizations, the total antibody levels in the serum of mice in the immunized group gradually increased, while no increase was observed in the control group (Figure 2C). The antibodies in the serum isolated two weeks after the last immunization were used to identify the IgG subtypes. As shown in Figure 2D, the IgG2b level was substantially higher than IgG1 and IgG3. The IgG2a level was also significantly higher than IgG1, indicating a prominent Th1 response.

### 3.3. TLA Specially Stimulated the Proliferation of Splenocyte in the Immunization Group

**Figure 3:**
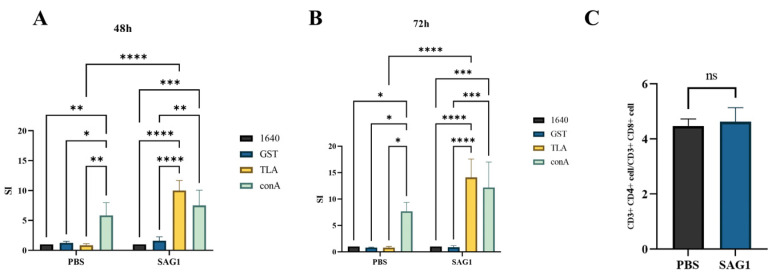
Spleen cell proliferation and T cell subtype analysis of immunized mice. (**A**) Spleen cell proliferation after 48 h of stimulation with TLA, ConA (positive control), GST (negative control), and 1640 (blank control). (**B**) Spleen cell proliferation after 72 h of stimulation with TLA, ConA (positive control), GST (negative control), and 1640 (blank control). (**C**) T cell subtype analysis by flow cytometry. Data for three mice in each group are displayed as mean ± SD (*n* = 3), with asterisks indicating statistical significance (* *p* < 0.05, ** *p* < 0.01, *** *p* < 0.001, **** *p* < 0.0001, and ns, no significance).

In mammals, infected macrophages and dendritic cells act as “Trojan horses” carrying *T. gondii* tachyzoites, facilitating their distribution within the host body. The spleen is a crucial peripheral immune organ that clears infected macrophages. CCK8 was utilized to assess the levels of splenocyte proliferation induced by TLA, ConA (positive control), GST (negative control), and 1640 (blank control). The proliferation SI at 48 h and 72 h post-stimulation was shown in Figure 3A,B. Significant splenocyte proliferation was observed in both TLA and ConA stimulation in the immunized group. Similar results were only observed in the control group in the ConA stimulation. This indicates that the immune cells in the spleen of immunized mice could rapidly and specifically recognize the TLA, contributing to host defense against *T. gondii* infection. However, the ratio of CD4^+^ T cells to CD8^+^ T cells did not alter significantly (Figure 3C).

### 3.4. Immunized Mice Showed Substantial Increases in Th1 (IFN-γ, IL-2, IL-12p70, and TNF-α) and Th2 (IL-10) Cytokine Levels

**Figure 4:**
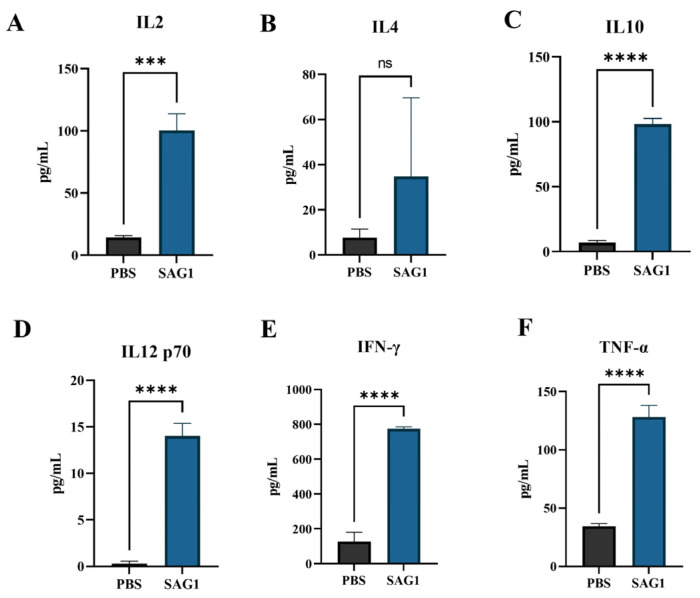
Cytokine levels produced by TLA-stimulated mouse splenocytes produced from spleens of three mice in each group. Cell-free supernatants were collected at 24 h for IL-2 (**A**) and IL-4 (**B**), 72 h for IL-10 (**C**) and TNF-α (**F**), and 96 h for IFN-γ (**E**) and IL-12p70 (**D**). The data are displayed as mean ± SD of three mice in each group (*** *p* < 0.001, **** *p* < 0.0001, and ns, no significance).

To identify the type of host T helper cell response induced by SAG1 protein immunization, the levels of Th1 and Th2-type cytokines released in culture supernatants after TLA stimulation of splenocytes were measured. The immunized group had significantly higher levels of Th1-type cytokines (IFN-γ, IL-2, IL-12p70, and TNF-α) and Th2-type cytokines (IL-10) than in the control group (Figure 4). The difference between the IL-4 levels in the immunized and the control groups was insignificant.

### 3.5. Full-Length SAG1 Recombinant Protein Immunization Enhanced Mouse Resistance to Lethal T. gondii Infection

**Figure 5:**
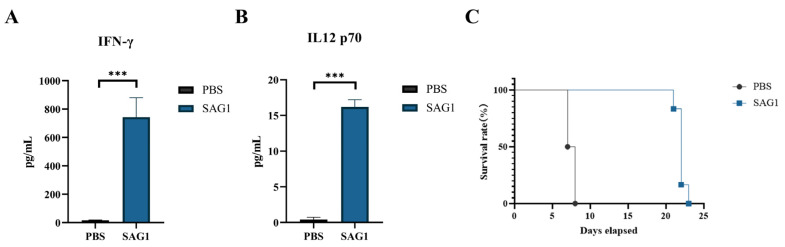
Survival time and cytokine levels in the serum of the immunized and control mice challenged with 50 *T. gondii* RH tachyzoites. (**A**) The IFN-γ levels in the mouse sera at seven days post-infection. (**B**) The IL-12 p70 levels in the mouse sera at seven days post-infection. (**C**) The survival time of challenged mice. Data of mice in each group are shown as mean ± SD *(n* = 3 in **A** and **B**, *n* = 6 in **C**), with asterisks indicating statistical significance (*** *p* < 0.001).

To assess the protective ability of the SAG1 recombinant protein, nine immunized mice and an equal number of control mice were, respectively, challenged with 50 tachyzoites of the *T. gondii* RH strain. In each group, six mice were used to monitor survival time, and the remaining three mice were sacrificed to detect cytokines in the serum on the seventh day post-challenge. As shown in Figure 5A, the Th1-typed cytokines in the infected mouse serum, including IL-12p70 and IFN-γ, were substantially higher than those in the control group. The survival time of all infected mice is shown in Figure 5B. In the control group, all infected mice died on the ninth day post-infection and showed some clinical manifestations, including mental exhaustion, slow movement, and decreased appetite before death. In contrast, in the immunized group, the mice started dying on the 22nd day post-infection, which is significantly longer than the control group. The average death time was increased by 14.50 ± 0.34 days (*p* < 0.0001), indicating that immunization with SAG1 recombinant protein substantially increases mouse resistance to lethal *T. gondii* infection.

## 4. Discussion

*T. gondii* vaccine development has been a key focus in preventing and controlling toxoplasmosis [27]. The subunit vaccine is a classic genetic engineering vaccine successfully used to prevent many infectious diseases [28,29,30,31,32]. About 30% of recombinant protein products on the market are produced through engineering *E. coli,* although this approach usually expresses insoluble recombinant protein, especially for eukaryotic proteins, which is not suitable for the preparation of vaccines. The SAG1 protein is undoubtedly considered the primary vaccine candidate because it is the tachyzoite major surface antigen with strong immunogenicity. However, several rare codons are present in the *SAG1* gene, and the native form of SAG1 has six disulfide bonds, making it difficult to express a soluble recombinant protein in prokaryotic systems. In the past, the native SAG1 protein purified from *T. gondii* tachyzoite by affinity chromatography and truncated recombinant SAG1 protein expressed by *Pichia pastoris* [33] or *E. coli* [34,35,36] have been used for *T. gondii* vaccine research. However, the purification process is difficult and time-consuming, and the truncated recombinant proteins usually lack the necessary epitopes. According to the IEBD database analysis, some MHC-II (Major histocompatibility complex, MHC) binding peptides have potential and are located at the C-terminal sequences of the SAG1 protein (Table S1). Previous studies have also demonstrated that B and T cell epitopes are mainly concentrated near the C-terminus of the SAG1 protein [37,38]. Godard et al. showed that the 238–256 amino acid residues of the SAG1 protein are essential T-cell epitopes [39]. In addition, Cardona et al. found that the SAG1 301–320 position exhibited the highest reactivity against human sera from patients infected with *T. gondii* [40]. In this study, the full-length CDS of the *SAG1* gene was amplified by PCR with special primers, and the complete and soluble SAG1 recombinant protein was obtained in *E. coli* Transetta (DE3) cells under the induced condition of 16 °C and 0.1 mM IPTG. This strain supplements rare codons of the SAG1 gene and is employed under low temperatures, which could contribute to the soluble expression of recombinant protein. We also noticed that the molecular weight of the recombinant protein was slightly larger than the native SAG1 in TLA (Figure 1D and Figure 2A). The reason was that to obtain the HIS-tag, an additional 36 amino acid residues were present at the N-terminal of the recombinant protein compared to the native SAG1 (Figure S1). Additionally, the theoretical molecular weight of the natural SAG1 protein is 34 kDa, while the recombinant protein is 38 kDa, whose positions in the gel were both slightly higher than their theoretical values (Figure 1D and Figure 2A)

Humoral immunity, characterized by antibodies, functions against *T. gondii* infection in at least three ways: opsonizing parasites for phagocytosis, blocking the tachyzoite invasion into cells, and activating the classical complement pathway. The SAG1 protein has been shown as a vital invasion-related ligand of *T. gondii* tachyzoites, and its monoclonal antibody could partially block the parasite invasion into host cells [41]. In the current study, the levels of polyclonal antibodies for full-length SAG1 recombinant protein significantly increased after the third immunization. After the fourth immunization, the titer of the polyclonal antibodies of SAG1 was significantly higher than the control group, which contributed to the host’s ability to resist *T. gondii* infection (Figure 2B). Furthermore, we also identified the subtype of IgG in the serum isolated from mice 14 days post-fourth immunization. The antigen-specific IgG2a and IgG2b levels were substantially higher than those of IgG1 and IgG3, indicating that SAG1 immunization induced both Th1- and Th2-type immunity, with the former being more prominent. Although humoral immunity can play a certain role in resisting *T. gondii* infection, cellular immunity is the major mechanism by which a vaccine enables the host to have a strong defense.

The innate immune cells, such as dendritic cells, macrophages, and neutrophils, migrate to the site of infection, where they detect the parasite. These cells recognize the parasite mainly through Toll-like receptors (TLRs) and secrete IL-12p70, which stimulates CD4^+^, CD8^+^ T, and natural killer cells to release IFN-γ. This cytokine plays an essential role in the host cell’s immune anti-*T. gondii* process intracellular proliferation [42]. When phagocytes are stimulated with IFN-γ, it effectively triggers several anti-parasitic mechanisms. These mechanisms include the upregulation of guanylate-binding proteins (GBPs) and immunity-related GTPases (IRGs), which help in attacking the parasitophorous vacuole (PV). Additionally, IFN-γ stimulation enhances antigen-presentation capabilities and upregulates the expression of other effective factors against parasites, such as reactive nitrogen and oxygen species. For non-hematopoietic cells, the upregulation of IRGs and GBPs also contributes to the destruction of the PV [43,44,45]. Additionally, TNF-α, IL-2, and IL-4 are protective against *T. gondii* infection. IL-4 and IL-10 are anti-inflammatory cytokines that help to minimize damage caused by an excessive immune inflammatory response. They also promote B cell differentiation and antibody production [41]. The spleen is an essential peripheral immunological organ that harbors many immune cells. It contributes significantly to the host’s resistance to pathogen infections.

In this investigation, compared to the control group, re-stimulation of spleen cells from the immunized mice resulted in the production of TNF-α, IFN-γ, IL-2, IL-10, and IL-12p70 but not IL-4. This suggests that immunization with the full-length SAG1 recombinant protein could selectively drive a response toward the Th1 type. To assess the efficacy of the full-length SAG1 recombinant protein against *T. gondii* infection, mice in the immunized and control groups were intraperitoneally inoculated with a lethal dose of *T. gondii* RH tachyzoites, as previously described. On day 7 post-inoculation, the IFN-γ and IL-12p70 levels in the serum of SAG1-immunized mice were substantially higher than those in the control group, which partially explains the reason that the immunized mice had stronger defenses against acute *T. gondii* infection. However, the IL-2, IL-4, IL-10, and TNF-α levels were undetectable in both groups, as they may be lower than the minimum detection limit. None of the mice in the control group survived beyond 9 days post-inoculation. In contrast, the immunized mice began dying on day 22 post-inoculation, with an average death time of 14.50 ± 0.34 days, which indicates our immune strategy has a certain effect on the resistance of mice to deadly infection caused by *T. gondii*.

Subunit vaccines only contain certain immune-active fragments of pathogens. While they may induce memory helper T and B cell production, they lack pathogen-associated molecular patterns (PAMPs). This makes it difficult to produce memory-killing T cells, resulting in low immune potency. Combining subunit vaccines with adjuvants could solve this problem. Adjuvants could cause local reactions at the vaccination site or contribute to other aspects of reactogenicity [46]. Immune adjuvants can serve as specific immune enhancers or carriers to target infected antigen-presenting cells (APCs), trigger innate immune responses, and enhance the immunogenicity of antigens. Aluminum adjuvants, Freund’s adjuvants, and propolis adjuvants are widely used. Moreover, it has been discovered in recent years that some small molecule peptides have the potential to boost the host’s immune response and have adjuvant properties. Furthermore, phagocytes and non-blood cell types respond to IFN-γ stimulation [40,41]. In this study, Freund’s adjuvant was chosen to enhance the immunogenicity of the SAG1 antigen. Both adjuvants are water in oil lotion that can carry antigens and stimulate the innate immune system. Complete Freund’s adjuvant includes heat-killed mycobacterial components in its structure, which can enhance the stimulation of immune response. Incomplete Freund’s adjuvant contributes to the sustained release of antigens, increases the half-life of antigens, and enhances local cellular immune responses. Although recombinant SAG1 protein with Freund’s adjuvant significantly prolonged the survival time of mice, the fate of infected mice remains unchanged. So far, there is almost no vaccine that could fully protect mice against lethal infection caused by *T. gondii* RH tachyzoite and this reason is still needed to be deeply explored.

## 5. Conclusions

In this study, the full-length and soluble SAG1 recombinant protein was acquired by *E. coli* Transetta (DE3) cells under the induced expression condition of 16 °C and 0.1 mM IPTG. Purified recombinant SAG1 protein substantially enhanced the resistibility of mice against *T. gondii* RH lethal infection by stimulating the host to produce robust cellular and humoral immunity. These results suggest that the full-length and soluble SAG1 recombinant protein could be a potential subunit vaccine candidate for toxoplasmosis.

## Data Availability

The datasets generated for this study are available on request to the corresponding author.

## Supplementary Materials

The following supporting information can be downloaded at: https://www.mdpi.com/article/10.3390/vaccines11111678/s1, Figure S1: Sequence alignment between recombinant SAG1 protein and native SAG1 proteins of *T. gondii* types I and II strains; Figure S2. The whole images of Figure 1B. Identification of SAG1 recombinant protein expressed in *E. coil* Transetta (DE3) cells by western blot; Figure S3. The whole images of Figure 1D. (D) Purified SAG1 recombinant protein identified by SDS-PAGE; Figure S4. The whole images of Figure 2A. Western blot analysis for native SAG1 in TLA with the experimental mouse sera; Table S1: Peptides with high score at C terminal of SAG1 protein binding to MHC class II molecules predicted by IEDB.
