# Supplementary material for: Boosting Mouse Defense against Lethal Toxoplasma gondii Infection with Full-Length and Soluble SAG1 Recombinant Protein
Source: Vaccines (Basel). 2023 Nov 2;11(11):1678. doi: 10.3390/vaccines11111678 (PMC10675489; doi:10.3390/vaccines11111678)
Supplement: Supplementary file 1 [file vaccines-11-01678-s001.zip › vaccines-2662587-supplementary.pdf]

Supplement materials

Boosting Mouse Defense against Lethal *Toxoplasma gondii* Infection with Full-Length and Soluble SAG1 Recombinant Protein

Xiang Li [1,2,†], Wei Yuan [1,2,†], Ting He [1,2], Ruiying Guo [1,2], Xiuxian Du [1,2], Yanhong He [1,2], Xuan Li [1,2], Saeed El-Ashram [3], Ebtesam M. Al-Olayan [4], Na Yang [1,2,*] and Xiaoyu Sang [1,2,*]

1 Key Laboratory of Livestock Infectious Diseases, Shenyang Agricultural University, Ministry of Education, Shenyang 110866, China; lx19980405@163.com (X.L.); yuanwei110923@163.com (W.Y.); 2021240709@stu.syau.edu.cn (T.H.); fruiming@stu.syau.edu.cn (R.G.); 2021220598@stu.syau.edu.cn (X.D.); 2022220595@stu.syau.edu.cn (Y.H.); 13081305895@163.com (X.L.)
2 College of Animal Science and Veterinary Medicine, Shenyang Agricultural University, Shenyang 110866, China
3 Zoology Department, Faculty of Science, Kafrelsheikh University, Kafr El-Sheikh 33516, Egypt; saeed_elashram@yahoo.com
4 Department of Zoology, College of Science, King Saud University, Riyadh 11451, Saudi Arabia; eolayan@ksu.edu.sa
* Correspondence: dayangna@syau.edu.cn (N.Y.); xysang2016@syau.edu.cn (X.S.)
† These authors contributed equally to this work.

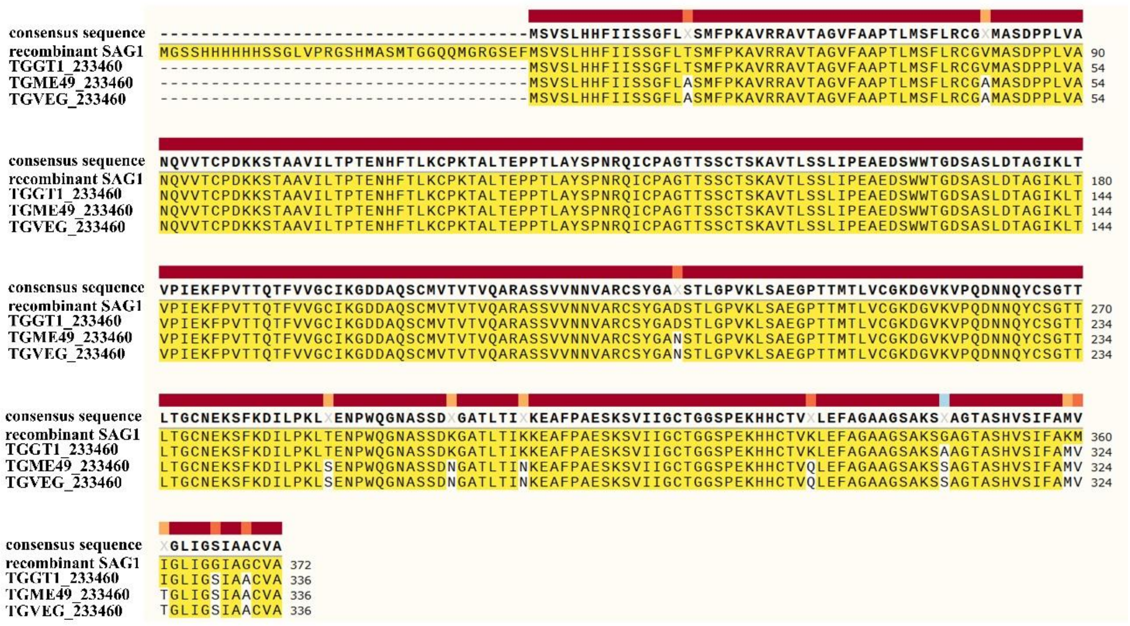

Figure S1. Sequence alignment between recombinant SAG1 protein and native SAG1 proteins of *T. gondii* types I and II strains. The amino acid sequence of the recombinant SAG1 was translated from the pET-28a-SAG1 sequence. The amino acid sequences of three native SAG1 were loaded from ToxoDB data.

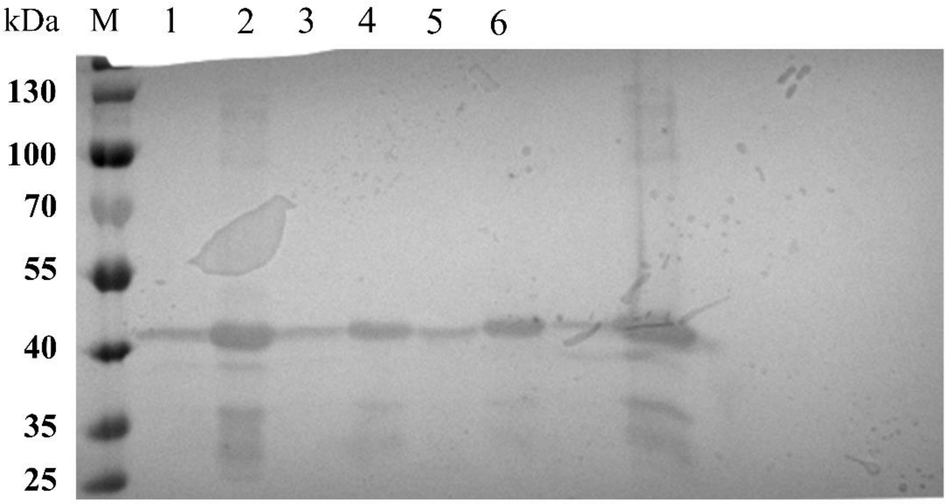

Figure S2. The whole images of Figure 1 B. Identification of SAG1 recombinant protein expressed in *E. coil* Transetta (DE3) cells by western blot. 1,3,5: The soluble protein of interest in the cell lysates supernatant from three randomly selected single colonies ; 2,4,6: The insoluble protein of interest in cell lysate precipitation from three randomly selected single colonies; M: Stander protein marker.

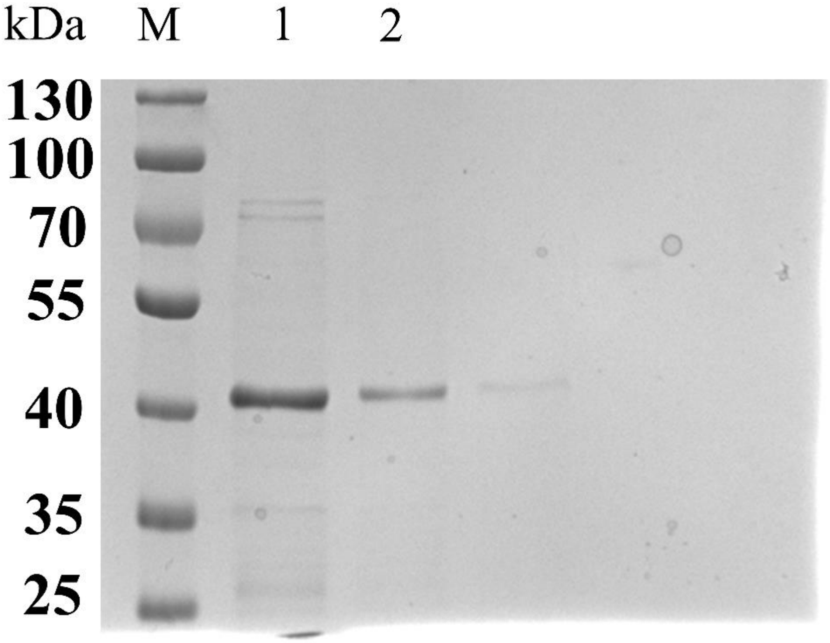

Figure S3. The whole images of Figure 1 D. (D) Purified SAG1 recombinant protein identified by SDS-PAGE. 1,2: Target protein was in eluent buffer; M: Stander protein marker.

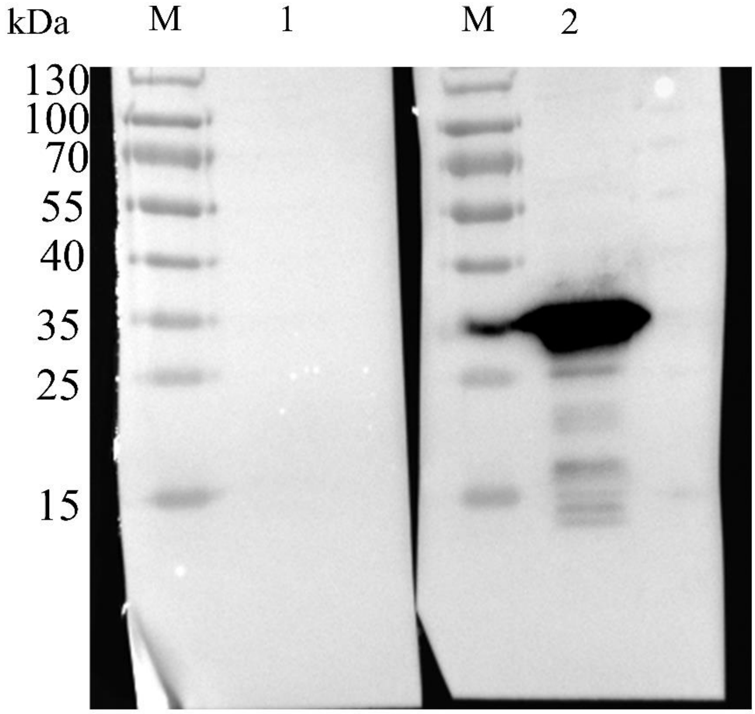

Figure S4. The whole images of Figure 2A. Western blot analysis for native SAG1 in TLA with the experimental mouse sera. 1:The reaction of TLA with the sera isolated from mice in the control group; 2:The reaction of TLA with the sera isolated from mice in the immunize group; M: Stander protein marker.

Table S1. Peptides with high score at C terminal of SAG1 protein binding to MHC class II molecules predicted by IEDB[a].

Allele[b]	Start	End	Peptide[c]	Score[d]
H2-IAd	175	189	TVQARASSVVNNVAR	0.519
**H2-IAd**	**308**	**322**	**AKSAAGTASHVSIFA**	**0.4901**
H2-IAd	171	185	MVTVTVQARASSVVN	0.4661
H2-IAd	296	310	TVKLEFAGAAGSAKS	0.4398
H2-IAd	169	183	SCMVTVTVQARASSV	0.4304
H2-IAd	170	184	CMVTVTVQARASSVV	0.4233
**H2-IAd**	**307**	**321**	**SAKSAAGTASHVSIF**	**0.4184**
H2-IAd	174	188	VTVQARASSVVNNVA	0.4077
H2-IAd	113	127	KAVTLSSLIPEAEDS	0.3972
**H2-IAd**	**302**	**316**	**AGAAGSAKSAAGTAS**	**0.3781**
H2-IAd	240	254	EKSFKDILPKLTENP	0.3711
H2-IAd	168	182	QSCMVTVTVQARASS	0.3714
**H2-IAd**	**295**	**309**	**CTVKLEFAGAAGSAK**	**0.3736**

**H2-IAd**	**306**	**320**	**GSAKSAAGTASHVSI**	**0.3635**
H2-IAd	297	311	VKLEFAGAAGSAKSA	0.3525
H2-IAd	63	77	KKSTAAVILTPTENH	0.3298
H2-IAd	61	75	PDKKSTAAVILTPTE	0.3298
**H2-IAd**	**301**	**315**	**FAGAAGSAKSAAGTA**	**0.3227**
H2-IAd	25	39	RAVTAGVFAAPTLMS	0.3234
H2-IAd	62	76	DKKSTAAVILTPTEN	0.3172

[a]The Immune Epitope Database (http://tools.immuneepitope.org/mhcii);
[b] H2-IAd alleles (mouse MHC class II molecules);
[c] 15 amino acids were chosen for analysis;
[d] High score indicates high level binding.
